# Sedentary bout durations and metabolic syndrome among working adults: a prospective cohort study

**DOI:** 10.1186/s12889-016-3570-3

**Published:** 2016-08-26

**Authors:** Takanori Honda, Sanmei Chen, Koji Yonemoto, Hiro Kishimoto, Tao Chen, Kenji Narazaki, Yuka Haeuchi, Shuzo Kumagai

**Affiliations:** 1 Department of Behavior and Health Sciences, Graduate School of Human-Environment Studies, Kyushu University, 6-1 Kasuga kouen, Kasuga City, Fukuoka Prefecture 816-8580 Japan; 2 Department of Epidemiology and Public Health, Graduate School of Medical Sciences, Kyushu University, 3-1-1 Maidashi, Higashi-ku, Fukuoka City, Fukuoka Prefecture 812-8582 Japan; 3 Research Fellow of the Japan Society for the Promotion of Science, 5-3-1 Kojimachi, Chiyoda-ku, Tokyo 102-0083 Japan; 4 Biostatistics Center, Kurume University, 67 Asahi-machi, Kurume, Fukuoka Prefecture 830-0011 Japan; 5 Department of Socio-Environmental Studies, Fukuoka Institute of Technology, 3-30-1 Wajiro-higashi, Higashi-ku, Fukuoka City, Fukuoka Prefecture 811-0295 Japan; 6 Faculty of Arts and Science, Kyushu University, 6-1 Kasuga kouen, Kasuga City, Fukuoka Prefecture 816-8580 Japan

**Keywords:** Epidemiology, Accelerometry, Sedentary lifestyle, Physical activity, Central obesity, Metabolic syndrome

## Abstract

**Background:**

This study aimed to examine the associations between time spent in prolonged and non-prolonged sedentary bouts and the development of metabolic syndrome.

**Methods:**

We used data from a prospective study of Japanese workers. Baseline examination was conducted between 2010 and 2011. A total of 430 office workers (58 women) aged 40-64 years without metabolic syndrome were followed up by annual health checkups until 2014. Metabolic syndrome was defined as having ≥ 3 out of 5 diagnostic criteria from the Joint Interim Statement 2009 definition. Sedentary time was assessed using a tri-axial accelerometer. Time spent in total, prolonged (accumulated ≥ 30 min) and non-prolonged sedentary bouts (accumulated < 30 min) was calculated. Cox proportional hazards models were used to estimate the risk of developing metabolic syndrome.

**Results:**

During a median follow-up of 3 years, 83 participants developed metabolic syndrome. After adjustment for age, sex, education, smoking, and family income, positive associations were observed between time spent in prolonged sedentary bouts and the development of metabolic syndrome. After additional adjustment for moderate-to-vigorous physical activity, those in the three highest quartiles of time spent in prolonged sedentary bouts showed higher risk of metabolic syndrome compared to the lowest quartile group, with adjusted hazard ratios (95 % confidence intervals) of 2.72 (1.30 – 5.73), 2.42 (1.11 – 5.50), and 2.85 (1.31 – 6.18), respectively. No associations were seen for time spent in total and non-prolonged sedentary bouts.

**Conclusions:**

Sedentary behavior accumulated in a prolonged manner was associated with an increased risk of metabolic syndrome. In devising public health recommendations for the prevention of metabolic disease, the avoidance of prolonged uninterrupted periods of sedentary behavior should be considered.

**Electronic supplementary material:**

The online version of this article (doi:10.1186/s12889-016-3570-3) contains supplementary material, which is available to authorized users.

## Background

Metabolic syndrome represents a cluster of metabolic disorders that include central obesity, elevated blood pressure, dyslipidemia, and hyperglycemia [1]. Metabolic syndrome is prevalent worldwide [2–4]; in the US, a recent report estimated that approximately one-fourth of adults have metabolic syndrome [2]. Since metabolic syndrome confers an elevated risk of cardiovascular diseases and type 2 diabetes [1], studies exploring modifiable risk factors for metabolic syndrome are essential to develop public health strategies for chronic disease prevention. A large body of epidemiological literature has shown that physical inactivity (i.e., lack of physical activity) is a driving factor for the global epidemic of non-communicable diseases [5].

Alongside physical inactivity, sedentary behavior, defined as prolonged periods of inactivity involving sitting or reclining, has recently been revealed to be associated with adverse metabolic and vascular health outcomes [6, 7]. Importantly, the detrimental effects of sedentary behavior on health are independent of lack of moderate-to-vigorous physical activity (MVPA) [8, 9]. Although a meta-analysis of cross-sectional studies revealed associations between sedentary behavior and metabolic syndrome [10], the role of sedentary behavior on the development of metabolic syndrome over time has been poorly understood. Only one study has reported a longitudinal association of sedentary behavior with metabolic syndrome; in that report, longer sedentary time was shown to increase the subsequent risk of developing metabolic syndrome independent of leisure-time physical activity [11]. The lack of evidence from prospective studies has precluded conclusions on the causality of the relationship between sedentary behavior and metabolic syndrome.

Recent studies have indicated that, in addition to total sedentary time, the manner in which sedentary time was accumulated has important health implications [12]. Several experimental studies have suggested that uninterrupted periods of sedentary behavior, compared to interrupted ones, exerted a detrimental impact on postprandial glucose and lipid responses [13, 14]. A cross-sectional observational study using an accelerometer reported that sedentary time in non-prolonged bouts was not associated with any cardiometabolic biomarkers, while that in prolonged bouts was associated with higher waist circumference and body mass index [15]. Other cross-sectional studies have even reported favorable associations between non-prolonged sedentary time and cardiometabolic and anthropometric measures [16, 17]. Accelerometers can objectively record minute-by-minute activities at different levels of intensity, and this objective measurement of physical activity allows us to capture periods (bouts) of consecutive minutes of activities. An analysis of accelerometry data showed that office workers spent 75 % of their workday being sedentary, with much of the time accumulated in prolonged (>30 min) bouts [18], suggesting that the health of office workers may be at risk of prolonged sedentary time and its consequences.

Thus far, to our knowledge, there have been no studies examining the associations between different durations of sedentary bouts and metabolic syndrome. We therefore addressed these issues by examining the prospective associations between objectively-measured time spent in sedentary behavior of different bout lengths and the development of metabolic syndrome. Here, we tested our hypothesis that a greater amount of sedentary time in prolonged bouts is associated with an increased risk of developing metabolic syndrome, independent of the levels of moderate-to-vigorous physical activity.

## Methods

### Participants

This study was conducted by using data from the Ryobi Health Survey, which is a prospective study carried out among Japanese workers to investigate social and behavioral risk factors for metabolic syndrome among working adults. The subjects were Japanese workers aged 30 years and over working in a Japanese enterprise group in Okayama prefecture, located on the western half of the main island of Japan. The enterprise, Ryobi Holdings, consists of seven companies involved primarily in information technology and transportation. Potential Subjects consisted of employees of the enterprises aged 40 to 64 years (*n* = 691) who were contacted by mail to participate in the Ryobi Health Survey. We did not include those aged between 30 and 39 in the present analyses since the Specific Health Checkups and Guidance in Japan (Tokutei Hoken Shido), a national screening and interventional program for metabolic syndrome, is geared toward those aged 40 and over. Baseline examination was conducted between January 2010 and March 2011, and the participants without metabolic syndrome at baseline were followed up in annual health checkups until March 2014.

Among the 691 subjects contacted by mail, 682 agreed to participate in the present study, representing a response rate of 98.7 %. Of this initial sample, data on the components of metabolic syndrome at baseline were available for 660 participants, and 502 participants without metabolic syndrome at baseline were eligible for the present study. Thirty-six participants without valid accelerometer data and 25 participants with missing data on covariates were further excluded. In addition, 11 individuals were lost to follow-up. Finally, 430 participants were included in the analyses. A comparison between subjects excluded from and those included in the present analysis is shown (see Additional file 1: Table S1). There was no evidence of selection bias due to the exclusion.

All participants provided written informed consent. This study was conducted in accordance with the Declaration of Helsinki. The entire study protocol was approved by the ethics committee of the Institute of Health Science, Kyushu University, Fukuoka, Japan.

### Assessment of metabolic syndrome

All data on anthropometry, blood pressure, lipid and glucose profile, and medication use were obtained from the annual health examinations, which were conducted in accordance with the Industrial Safety and Health Act [19]. Height and body weight were measured in light clothing without shoes. Waist circumference was measured to the nearest 0.1 cm at the umbilical level while standing. Systolic and diastolic blood pressures were measured at rest by an automated sphygmomanometer. Serum triglycerides, high-density lipoprotein (HDL) cholesterol, and blood glucose were measured using enzymatic methods. All participants were asked to fast overnight before the blood test.

Metabolic syndrome was defined based on the Joint Interim Statement 2009 definition [1]. Specifically, participants having ≥ 3 of the following five clinical measures were considered as having metabolic syndrome: (1) central obesity (waist circumference (≥90 cm in men, or ≥ 85 cm in women); (2) elevated blood pressure, defined as systolic blood pressure ≥ 130 mmHg or diastolic blood pressure ≥ 85 mmHg, or taking an antihypertensive medication; (3) elevated fasting blood glucose level ≥ 5.6 mmol/L or taking a hypoglycemic medication; (4) decreased HDL-cholesterol level (<1.0 mmol/L in men or < 1.3 mmol/L in women); and (5) hypertriglyceridemia (≥1.7 mmol/L) or taking a lipid-lowering medication.

### Objective measurement of sedentary behavior and physical activity

Sedentary behavior and MVPA were assessed using a tri-axial accelerometer device (Active style Pro HJA 350-IT; Omron Healthcare Co., Ltd., Kyoto, Japan). Details of the accelerometer measurement procedure have been reported elsewhere [20]. Briefly, participants wore the device during waking hours for 10 consecutive days, except while bathing or sleeping. Data were recorded in 60-sec epochs. The accuracy of the intensity estimation has been validated with the Douglas bag method [21, 22]. Non-wear time was defined as a period of at least 60 consecutive minutes of no activity (i.e., estimated activity intensity < 1.0 metabolic equivalent, or MET) with allowance for up to two consecutive minutes of activities with intensity equal to 1.0 MET. We adapted the SAS macro program for the ActiGraph monitor provided by the National Cancer Institute to compute daily non-wear time [23], with modifications for our accelerometer [20]. Days with at least 600 min of wear time were considered valid [24]. Participants with at least four valid days were included in the analysis.

In this study, sedentary behavior was defined as any activity with an accelerometer-estimated intensity of ≤ 1.5 METs. We considered each minute that the activity intensity was ≤ 1.5 METs as sedentary time. A sedentary bout was defined as a period of time in continuous sedentary time where the activity intensity fell into the sedentary range with no interruption. For example, a bout of 30 min of sedentary time was not an accumulation of three 10-min bouts but rather a consecutive 30-min period of sedentary time. The amounts of time spent in prolonged (accumulated ≥ 30 min) and non-prolonged sedentary bouts (accumulated < 30 min) were calculated separately. Total sedentary time was calculated as the sum of the prolonged and non-prolonged sedentary periods.

MVPA was defined as activities of ≥ 3 METs. An MVPA bout was defined as a period of time in continuous activities where the activity intensity was ≥ 3 METs. A bout of MVPA lasting for at least 10 min, with allowance for up to 2 min of non-MVPA activity, was considered an MVPA period, which is consistent with the consensus recommendation that physical activity accumulated in periods lasing for ≥ 10 min benefits health [25].

### Other variables

Information on education (higher or lower than university education) and current smoking habits (yes or no) was obtained from a self-report questionnaire. Household income data were obtained by questionnaire and categorized as <4 million, 4-8 million, or ≥ 8 million Japanese yen per year.

### Statistical analysis

All statistical analyses were performed with the SAS software version 9.3 (SAS Institute, Cary, NC, USA) with a significance level of α = 0.05. Person-time for each participant was calculated from the date of baseline examination to the date of the first occurrence of metabolic syndrome or the last examination, whichever came first. Only two participants missed a follow-up examination and were confirmed as having metabolic syndrome in the subsequent year. Since the findings were basically unchanged after excluding these participants, results were presented including data from these participants.

Given that the wear-time potentially influences the sedentary time, sedentary time variables were adjusted for wear-time using the residual method [26, 27]. Wear time-adjusted sedentary time variables were divided into sex-specific quartiles for analyses. The quartile boundaries for each exposure are shown (see Additional file 1: Table S2). The variables were expressed as the median (interquartile range, IQR) for continuous data, and the frequency for categorical data.

To model the effects of sedentary variables on first-ever metabolic syndrome, Cox proportional hazards models were used to calculate hazard ratios (HRs) with 95 % confidence intervals (CIs) for the development of metabolic syndrome by quartiles of total, prolonged and non-prolonged sedentary time. The proportional hazards assumption was assessed by visual inspection of log-log plots. The first model was adjusted for sex and age. The second model was adjusted for sex, age, education, and current smoking. To examine whether the associations of sedentary time with metabolic syndrome were independent of physical activity, the third model was adjusted for variables in the second model and MVPA. Waist circumference was further adjusted in the full model to examine whether the associations were independent of central obesity. We tested interactions of sedentary variables with age, sex, and MVPA (<150 or ≥ 150 min/week) in each survival model, to examine potential effect-modifications by age, sex, and physical activity. None of the interaction terms were statistically significant, showing that these factors did not modify the effect of sedentary behavior on the development of metabolic syndrome. Here, we should note that metabolic syndrome is a “reversible” state. To elucidate the potential impact of this reversibility on the Cox proportional hazard model, we repeated the analysis while excluding those who took medications at baseline (we assumed that these subjects would be more likely to receive treatment or lifestyle intervention by physicians during the follow-up period). The result was not substantially changed from the main analysis, suggesting that such cases would not have affected the present findings, and thus we presented the results drawn from the whole sample.

Sensitivity analyses were performed using different cut-off points (≥10 and ≥ 20 min) for differentiating prolonged sedentary time from non-prolonged sedentary time. Additionally, the associations between sedentary variables and metabolic syndrome were analyzed in those at a higher risk of developing metabolic syndrome (with two affected components at baseline) and those with a lower risk (with one or no affected components) separately.

## Results

### Baseline characteristics of the study participants

Among the 430 participants, 87.5 % were men and the median age at baseline was 48 years. The participants wore the accelerometer for 8.5 valid days on average, and the median (IQR) device wear-time was 846 (786 – 920) minutes/day. Table 1 summarizes participants’ baseline characteristics by quartiles of prolonged sedentary time. Participants who had higher amounts of prolonged sedentary time were younger and more highly educated, and were less likely to be current smokers. There was a significant difference in household income among groups.

**Table 1:** Sample characteristics by quartiles of time spent in prolonged sedentary bouts (≥30 min), the Ryobi Health Survey 2009 – 2010 (*n* = 430)

Characteristics	Time spent in prolonged sedentary bouts^a^								*p* for trend
	Q1 (*n* = 107)		Q2 (*n* = 108)		Q3 (*n* = 107)		Q4 (*n* = 108)		
Age group, % (n)									<0.001
40–49	48.6	(52)	45.4	(49)	62.6	(67)	76.9	(83)	
50–59	40.2	(43)	41.7	(45)	28.0	(30)	20.4	(22)	
60–64	11.2	(12)	13.0	(14)	9.3	(10)	2.8	(3)	
Women, % (n)	13.1	(14)	13.9	(15)	13.1	(14)	13.9	(15)	0.9130
Education, college or university level, % (n)	36.4	(39)	57.4	(62)	72.0	(77)	73.1	(79)	<0.001
Current smoker, % (n)	36.4	(39)	38.9	(42)	29.0	(31)	25.0	(27)	0.028
Family income (JPY), % (n)									0.028
< 4 million	19.6	(21)	20.4	(22)	5.6	(6)	2.8	(3)	
4–8 million	57.9	(62)	49.1	(53)	65.4	(70)	74.1	(80)	
8+ million	22.4	(24)	30.6	(33)	29.0	(31)	23.1	(25)	
Moderate-to-vigorous physical activity, min/wk, median (interquartile range)	50	(11, 141)	66	(23, 168)	57	(11, 151)	41	(11, 174)	0.564
Device wear-time, min/d, median (interquartile range)	850	(785, 916)	843	(790, 922)	834	(787, 912)	865	(776, 929)	0.7503
Central obesity, % (n)	14.0	(15)	11.1	(12)	13.1	(14)	11.1	(12)	0.635
Elevated blood pressure, % (n)	29.0	(31)	33.3	(36)	28.0	(30)	32.4	(35)	0.801
Hypertriglyceridemia, % (n)	10.3	(11)	18.5	(20)	16.8	(18)	24.1	(26)	0.015
Low HDL-cholesterol level, % (n)	1.9	(2)	2.8	(3)	4.7	(5)	1.9	(2)	0.799
Hyperglycemia, % (n)	40.2	(43)	35.2	(38)	32.7	(35)	25.9	(28)	0.026
Number of affected components, % (n)^b^									0.878
Zero	30.8	(33)	29.6	(32)	33.6	(36)	27.8	(30)	
One	43.0	(46)	39.8	(43)	37.4	(40)	49.1	(53)	
Two	26.2	(28)	30.6	(33)	29.0	(31)	23.1	(25)	

Data are presented as a median (interquartile range) or % (n). HDL-cholesterol, high-density lipoprotein cholesterol

^a^Time spent in prolonged sedentary time was adjusted for time spent wearing the device using the residual method prior to classifying into sex-specific quartiles (Q1 – Q4). Cut-offs for quartiles were 106.7, 165.5, and 269.2 min/day for men, and 65.1, 122.7, and 195.4 for women for those who wore the accelerometer device for the average amount of wear-time

^b^Number of components of metabolic syndrome at baseline survey

### Prospective effects of baseline sedentary behavior on the risk of metabolic syndrome

During a median follow-up period of 3 years (IQR 3-4 years), 76 men and 7 women developed metabolic syndrome. The HRs (95 % CIs) of total, non-prolonged, and prolonged sedentary time for metabolic syndrome are shown in Table 2. No associations between total sedentary time and metabolic syndrome were found in any models. Similarly, the association of non-prolonged sedentary time (<30-min bouts) with the development of metabolic syndrome was not significant in any models. On the other hand, significant associations were observed between prolonged sedentary time (≥30-min bouts) and increased risk of metabolic syndrome. Those in the second and the higher quartiles showed significantly higher risk of metabolic syndrome compared with the lowest quartile group in the sex and age-adjusted and multivariate-adjusted models. After adjustment for MVPA, the association remained significant with adjusted HRs (95 % CI) of 2.72 (1.30 – 5.73), 2.42 (1.11 – 5.5), and 2.85 (1.31 – 6.18). This association did not change even after adjustment for waist circumference. When prolonged sedentary time was defined as ≥ 10-min or ≥ 20-min bouts, neither prolonged nor non-prolonged sedentary time was associated with increased risk of metabolic syndrome (Additional file 1: Table S3).

**Table 2:** Multivariable-adjusted hazard ratios (95 % confidence intervals) for the development of metabolic syndrome, the Ryobi Health Survey 2009 – 2010 (*n* = 430)

	Cases (n)	Incident rate (per 1000 person-years)	Model 1			Model 2			Model 3			Model 4		
			HR	95 % CI	*p* value	HR	95 % CI	*p* value	HR	95 % CI	*p* value	HR	95 % CI	*p* value
Total sedentary time (≥1-min bout)														
Q1	22	62.5	1.00			1.00			1.00			1.00		
Q2	23	68.0	1.23	(0.63 – 2.39)	0.542	1.39	(0.67 – 2.86)	0.376	1.37	(0.66 – 2.82)	0.398	1.50	(0.73 – 3.09)	0.272
Q3	20	63.3	1.66	(0.88 – 3.13)	0.116	1.87	(0.94 – 3.72)	0.075	1.84	(0.92 – 3.68)	0.083	1.76	(0.87 – 3.55)	0.118
Q4	18	56.6	1.12	(0.56 – 2.21)	0.752	1.30	(0.61 – 2.76)	0.500	1.26	(0.59 – 2.69)	0.559	1.55	(0.70 – 3.43)	0.278
Non-prolonged sedentary time (<30-min bout)														
Q1	16	52.6	1.00			1.00			1.00			1.00		
Q2	18	55.9	0.71	(0.38 – 1.31)	0.268	0.72	(0.39 – 1.34)	0.298	0.71	(0.38 – 1.33)	0.287	0.79	(0.42 – 1.48)	0.465
Q3	27	81.3	0.82	(0.45 – 1.48)	0.500	0.82	(0.45 – 1.51)	0.520	0.81	(0.44 – 1.49)	0.491	1.09	(0.59 – 2.03)	0.785
Q4	22	60.1	0.85	(0.47 – 1.52)	0.573	0.85	(0.47 – 1.56)	0.606	0.83	(0.45 – 1.52)	0.546	1.08	(0.57 – 2.02)	0.817
Prolonged sedentary time (≥30-min bout)														
Q1	20	58.3	1.00			1.00			1.00			1.00		
Q2	23	70.6	2.58	(1.24 – 5.37)	0.011	2.71	(1.29 – 5.68)	0.009	2.72	(1.30 – 5.73)	0.008	3.03	(1.42 – 6.49)	0.004
Q3	21	64.4	2.16	(1.02 – 4.59)	0.045	2.41	(1.11 – 5.25)	0.026	2.42	(1.11 – 5.25)	0.026	2.25	(1.03 – 4.92)	0.040
Q4	19	57.8	2.49	(1.18 – 5.24)	0.017	2.86	(1.31 – 6.21)	0.008	2.85	(1.31 – 6.18)	0.008	2.90	(1.30 – 6.44)	0.009

*HR* hazard ratio, *CI* confidence interval. Sedentary variables were adjusted for time spent wearing the device using the residual method prior to classifying into sex-specific quartiles. Model 1 was adjusted for sex and age. Model 2 was adjusted for sex, age, education, smoking, and family income. Model 3 was additionally adjusted for moderate-to-vigorous physical activity. Model 4 was additionally adjusted for waist circumference. Cut-offs for quartiles were 106.7, 165.5, and 269.2 min/day for men, and 65.1, 122.7, and 195.4 for women among those who wore the accelerometer device for the average amount of wear-time

**Fig. 1:**
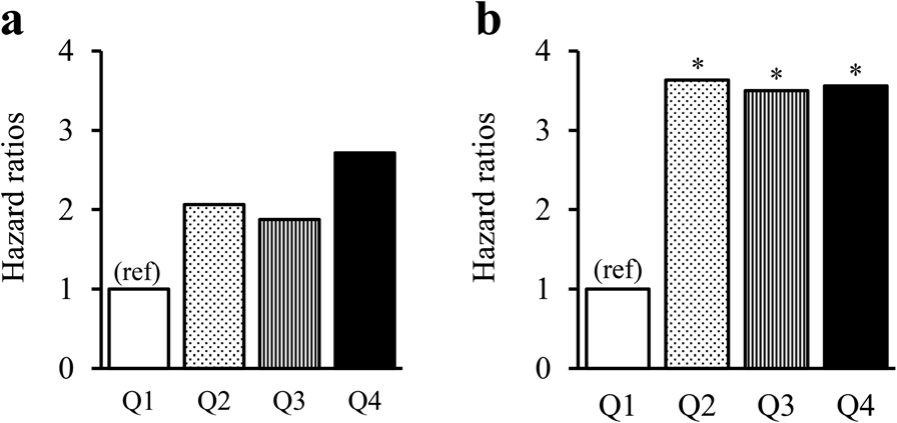
Associations of time spent in prolonged sedentary bouts with metabolic syndrome according to the number of metabolic syndrome components at baseline. Panel **a**, Participants who at baseline had zero or one affected component. The numbers of participants in each group were 79, 75, 76, and 83 for Q1 to Q4 in panel A, respectively. Panel **b**, Participants with two affected components at baseline. The numbers of participants in each group were 28, 33, 31, and 25 for Q1 to Q4 in panel B, respectively. *p < 0.05

Figure 1 illustrates the associations of prolonged sedentary time (≥30-min bouts) with metabolic syndrome by the number of components of metabolic syndrome at baseline. In the group with 0-1 metabolic syndrome components, although the multivariable-adjusted hazard rates in the three highest quartiles were 1.9 to 2.7-fold greater than that in the lowest group, these differences were not statistically significant (panel A). In contrast, among participants with two affected components, the three highest quartile groups had about 3.5-fold significantly greater risk of developing metabolic syndrome compared with the lowest quartile group (panel B).

## Discussion

In this longitudinal study of adult office workers, we found that longer time spent in prolonged sedentary bouts (≥30-min bouts), but not in shorter ones, was significantly associated with higher risk of metabolic syndrome. The significance did not change after additional adjustment for MVPA, suggesting that the associations between time spent in prolonged sedentary bouts and metabolic syndrome was independent of MVPA.

There were several strengths in the present study. To our knowledge, this is the first study to examine prospective associations between objectively-measured sedentary time and metabolic syndrome. The prospective design allowed to establish a temporal sequence. Secondly, the use of a tri-axial accelerometer with a validated algorithm for estimating low-intensity physical activities enhanced the accuracy of our assessment. In addition, the minute-by-minute monitoring approach enabled us to quantify sedentary periods of prolonged and non-prolonged bout lengths.

Our study also has several limitations. First, the results may not be generalizable to other working adult populations as the participants were mainly engaged in sedentary occupations. Second, sedentary time was assessed only at baseline, which could have led to misclassification. Such misclassification, if present, would have weakened the relationship between sedentary time and the development of metabolic syndrome, and biased the results toward the null hypothesis. Third, the accelerometer is unable to differentiate standing and sitting. Indeed, we observed that quiet standing could be incorrectly classified as a sedentary period by the accelerometer in a laboratory setting (data not shown). Future research should use an objective measure which is able to distinguish sitting from standing postures. However, we would expect that most office workers would spend a majority of their work time sitting rather than standing, and few occupations require standing quietly for half of an hour or more. Finally, we did not measure some important covariates, such as diet and family history of type 2 diabetes.

The findings from the present study suggested that objectively-measured sedentary time is an independent risk factor for metabolic syndrome, which was consistent with a previous longitudinal observation using a self-reported measure of sedentary behaviors [11]. Also, our findings were in line with cross-sectional studies using an accelerometer device to assess sedentary time [28–30], although some exceptions exist [31]. Our findings have extended these works by examining prolonged sedentary time and metabolic syndrome over time. Similarly, in a cross-sectional study of overweight/obese adults it was reported that prolonged sedentary time, but not non-prolonged sedentary time (<30-min bouts), was associated with cardio-metabolic risks [15]. Indeed, another study even found favorable associations between sedentary time in very short bouts (representing frequent transitions of postures) and cardio-metabolic risk profiles [16]. There has also been a cross-sectional study which reported no associations between sedentary time and the presence of metabolic syndrome [31]. The discrepancy may be partly attributable to methodological issues. Typically, sedentary time has been quantified by counting every single minute (i.e., ≥ 1-min bouts) in which the activity counts were below a threshold for sedentary behavior [29, 30]. By this method, sedentary time would be compounded not only by prolonged but also non-prolonged sedentary periods; thus the effects on metabolic syndrome would partly cancel each other out, which would presumably be inappropriate to reflect the prolonged nature of sedentary behavior. To our knowledge, only one study examining metabolic syndrome employed sedentary bouts (≥5-min) to calculate time spent in sedentary behavior [28]; however, a period of 5 min now seems to be insufficient for a definition of sustained sedentary behavior, based on our present results suggesting that prolonged sedentary time of 30 min contributes to the development of metabolic syndrome.

In the present analysis, the three highest quartiles of prolonged sedentary time had similar HRs, suggesting that there was a threshold of duration of prolonged sedentary time. Given that the residual method was used to adjust for the device wear-time, the cut-off value between the lowest and second quartiles (106.7 min for men and 65.1 min for women) should be interpreted as applying to individuals wearing the device for the average wear-time of this population. These values could be underestimated and thus conservative compared to real-world settings, considering that, in practice, the device wear-time is likely to be shorter than actual waking hours.

We also observed that the associations were stronger in participants with two affected components at baseline compared with those with no or one affected component, suggesting that those at higher metabolic risk could benefit more from a reduction in prolonged sedentary behavior. However, the absence of a significant association among those with no or one component does not necessarily indicate that the recommendation to reduce prolonged sedentary periods should be withheld from this population. Due to the short follow-up periods, only 32 cases of metabolic syndrome occurred in this group, which could potentially have resulted in insufficient statistical power to detect to a significant difference. Further investigation, including studies with longer follow-up, is needed to address this issue.

The mechanisms by which sedentary behavior independently increases the risk of chronic disease remain to be fully elucidated. Our results showed no substantial change after adjustment for waist circumference, suggesting that mechanisms other than central obesity may contribute to the deleterious impact of sedentary behavior on metabolic syndrome. Hamilton and colleagues suggested that the activities of lipoprotein lipase, which locally regulate the uptake of triglycerides into muscle and the HDL-cholesterol concentration, were suppressed by prolonged periods without muscle contraction [32, 33]. Bed rest, a model of extreme sedentary behavior, has been shown to induce insulin resistance in skeletal muscle, reduced fatty acid oxidation, muscle atrophy, and a shift in muscle fiber type and ectopic fat storage [34]. Those physiological adaptations could also be induced by a certain prolonged period of sedentary time (i.e., ≥ 30 min).

Our findings suggest the need for public health messages and policies to reduce not total but sustained sedentary periods, which has not yet been considered [35]. Interruptions of sedentary bouts in the present analysis were probably made by standing up or walking or by movement during sitting in which the intensity exceeded 1.5 METs. Therefore, not only the use of brief activity breaks to disrupt prolonged periods of sitting but also increasing movements of ≥ 1.5 METs while sitting, such as stretching, may be beneficial for prevention of metabolic syndrome.

## Conclusions

Sedentary behavior accumulated in a prolonged manner was shown to be associated with an increased risk of developing metabolic syndrome. Reducing time spent in prolonged sedentary bouts may be beneficial for the prevention of metabolic syndrome. These results highlight the importance of sedentary bouts, which should be taken into account in the recommendations for the primary prevention of metabolic syndrome. Public health recommendations regarding the prevention of metabolic diseases may need to include avoiding prolonged uninterrupted periods.

## Additional file

Additional file 1: Table S1. Comparisons between included and excluded subjects. **Table S2.** Quartile boundaries for time spent in total, non-prolonged and prolonged sedentary bouts. **Table S3.** Multivariable-adjusted hazard ratios (95 % confidence intervals) for the development of metabolic syndrome by different bout thresholds. (DOCX 23 kb)

## Abbreviations

CIs: Confidence intervals
HDL-cholesterol: High-density lipoprotein cholesterol
HRs: Hazard ratios
IQR: Interquartile range
MET: Metabolic equivalent
MVPA: Moderate-to-vigorous physical activity
