# Supplementary material for: Sedentary bout durations and metabolic syndrome among working adults: a prospective cohort study
Source: BMC Public Health. 2016 Aug 26;16(1):888. doi: 10.1186/s12889-016-3570-3 (PMC5000401; doi:10.1186/s12889-016-3570-3)
Supplement: Additional file 1: Table S1. — Comparisons between included and excluded subjects. Table S2. Quartile boundaries for time spent in total, non-prolonged and prolonged sedentary bouts. Table S3. Multivariable-adjusted hazard ratios (95 % confidence intervals) for the development of metabolic syndrome by different bout thresholds. (DOCX 23 kb) [file 12889_2016_3570_MOESM1_ESM.docx]

Table S1. Comparisons between included and excluded subjects.

|  | Number of missing | Included | | Excluded | | p value |
| --- | --- | --- | --- | --- | --- | --- |
| Age group, % (n) | 0 |  |  |  |  | 0.4353 |
| 40-49 |  | 58.4 | (251) | 54.2 | (39) |  |
| 50-59 |  | 32.6 | (140) | 31.9 | (23) |  |
| 60+ |  | 9.1 | (39) | 13.9 | (10) |  |
| Women, % (n) | 0 | 13.5 | (58) | 11.1 | (8) | 0.581 |
| Education, college or university level, % (n) | 15 | 59.8 | (257) | 47.4 | (27) | 0.074 |
| Current smoker, % (n) | 7 | 32.3 | (139) | 35.4 | (23) | 0.624 |
| Family income (JPY), % (n) | 10 |  |  |  |  | 0.629 |
| <4 million |  | 12.1 | (52) | 15.2 | (9) |  |
| 4-8 million |  | 61.6 | (265) | 63.6 | (40) |  |
| 8+ million |  | 26.3 | (113) | 21.2 | (13) |  |
| Moderate-to-vigorous physical activity, min/wk, median (interquartile range) | 12 | 57 | (12, 165) | 37 | (0, 174) | 0.224 |
| Central obesity, % (n) | 0 | 12.3 | (53) | 11.1 | (8) | 0.770 |
| Elevated blood pressure, % (n) | 0 | 30.7 | (132) | 27.8 | (20) | 0.618 |
| Hypertriglyceridemia, % (n) | 0 | 17.4 | (75) | 12.5 | (9) | 0.299 |
| Low HDL-cholesterol level, % (n) | 0 | 2.8 | (12) | 1.4 | (1) | 0.488 |
| Hyperglycemia, % (n) | 0 | 33.5 | (144) | 27.8 | (20) | 0.339 |
| Number of affected components, % (n) | 0 |  |  |  |  | 0.245 |
| zero |  | 30.5 | (131) | 38.9 | (28) |  |
| one |  | 42.3 | (182) | 41.7 | (30) |  |
| two |  | 27.2 | (117) | 19.4 | (14) |  |

Data are presented as a median (interquartile range) or % (n).

Table S2. Quartile boundaries for time spent in total, non-prolonged and prolonged sedentary bouts.

|  | Men | | | Women | | |
| --- | --- | --- | --- | --- | --- | --- |
|  | 25 percentile | 50 percentile | 75 percentile | 25 percentile | 50 percentile | 75 percentile |
| Total sedentary time (≥ 1-min bout) | 445.7 | 536.4 | 604.2 | 407.5 | 506.2 | 575.9 |
| Non-prolonged sedentary time (< 30-min bout) | 276.7 | 327.9 | 375.5 | 295.9 | 348.0 | 391.0 |
| Prolonged sedentary time (≥ 30-min bout) | 106.7 | 165.5 | 269.2 | 65.1 | 122.7 | 195.4 |

Values were calculated after adjusted for wear time using the residual method.

Table S3. Multivariable-adjusted hazard ratios (95% confidence intervals) for the development of metabolic syndrome by different bout thresholds.

|  | Cases (n) | Incident rate (per 1,000 person-years) | Model 1 | | | Model 2 | | | Model 3 | | | Model 4 | | |
| --- | --- | --- | --- | --- | --- | --- | --- | --- | --- | --- | --- | --- | --- | --- |
|  |  |  | HR | 95% CI | p value | HR | 95% CI | p value | HR | 95% CI | p value | HR | 95% CI | p value |
| Defined with a 10-min threshold | | |  |  |  |  |  |  |  |  |  |  |  |  |
| Non-prolonged sedentary time (< 10-min bout) | | |  |  |  |  |  |  |  |  |  |  |  |  |
| Q1 | 22 | 62.5 | 1.00 |  |  | 1.00 |  |  | 1.00 |  |  | 1.00 |  |  |
| Q2 | 23 | 68.0 | 1.08 | (0.6 - 1.94) | 0.800 | 1.06 | (0.59 - 1.92) | 0.840 | 1.06 | (0.59 - 1.91) | 0.860 | 0.99 | (0.55 - 1.79) | 0.970 |
| Q3 | 20 | 63.3 | 1.00 | (0.54 - 1.83) | 1.000 | 0.98 | (0.53 - 1.81) | 0.960 | 0.96 | (0.52 - 1.77) | 0.890 | 1.05 | (0.57 - 1.95) | 0.880 |
| Q4 | 18 | 56.6 | 0.90 | (0.48 - 1.68) | 0.730 | 0.88 | (0.46 - 1.66) | 0.680 | 0.85 | (0.45 - 1.62) | 0.620 | 0.78 | (0.4 - 1.51) | 0.460 |
| Prolonged sedentary time (≥ 10-min bout) | | |  |  |  |  |  |  |  |  |  |  |  |  |
| Q1 | 16 | 52.6 | 1.00 |  |  | 1.00 |  |  | 1.00 |  |  | 1.00 |  |  |
| Q2 | 18 | 55.9 | 1.06 | (0.54 - 2.08) | 0.870 | 1.15 | (0.56 - 2.35) | 0.700 | 1.15 | (0.56 - 2.34) | 0.710 | 1.27 | (0.61 - 2.64) | 0.520 |
| Q3 | 27 | 81.3 | 1.63 | (0.87 - 3.05) | 0.130 | 1.85 | (0.94 - 3.67) | 0.080 | 1.84 | (0.93 - 3.65) | 0.080 | 1.88 | (0.94 - 3.75) | 0.070 |
| Q4 | 22 | 60.1 | 1.23 | (0.63 - 2.39) | 0.550 | 1.41 | (0.68 - 2.91) | 0.350 | 1.40 | (0.68 - 2.89) | 0.360 | 1.70 | (0.81 - 3.06) | 0.160 |
| Defined with a 20-min threshold | | | |  |  |  |  |  |  |  |  |  |  |  |
| Non-prolonged sedentary time (< 20-min bout) | | | |  |  |  |  |  |  |  |  |  |  |  |
| Q1 | 20 | 58.3 | 1.00 |  |  | 1.00 |  |  | 1.00 |  |  | 1.00 |  |  |
| Q2 | 23 | 70.6 | 1.23 | (0.67 - 2.23) | 0.510 | 1.23 | (0.67 - 2.25) | 0.500 | 1.22 | (0.67 - 2.23) | 0.520 | 1.27 | (0.69 - 2.32) | 0.450 |
| Q3 | 21 | 64.4 | 1.10 | (0.59 - 2.02) | 0.770 | 1.11 | (0.60 - 2.07) | 0.740 | 1.10 | (0.59 - 2.06) | 0.760 | 1.26 | (0.68 - 2.37) | 0.460 |
| Q4 | 19 | 57.8 | 1.00 | (0.53 - 1.87) | 0.990 | 1.00 | (0.53 - 1.89) | 1.000 | 0.98 | (0.52 - 1.85) | 0.940 | 1.11 | (0.58 - 2.12) | 0.750 |
| Prolonged sedentary time (≥ 20-min bout) | | | |  |  |  |  |  |  |  |  |  |  |  |
| Q1 | 15 | 48.9 | 1.00 |  |  | 1.00 |  |  | 1.00 |  |  | 1.00 |  |  |
| Q2 | 19 | 59.7 | 1.22 | (0.62 - 2.41) | 0.560 | 1.25 | (0.62 - 2.53) | 0.530 | 1.25 | (0.62 - 2.54) | 0.530 | 1.42 | (0.69 - 2.93) | 0.340 |
| Q3 | 27 | 81.3 | 1.75 | (0.92 - 3.31) | 0.090 | 1.92 | (0.98 - 3.78) | 0.060 | 1.91 | (0.97 - 3.76) | 0.060 | 1.85 | (0.94 - 3.65) | 0.080 |
| Q4 | 22 | 59.9 | 1.32 | (0.67 - 2.59) | 0.430 | 1.47 | (0.72 - 3.02) | 0.290 | 1.46 | (0.71 - 3.01) | 0.300 | 1.48 | (0.70 - 3.10) | 0.300 |

Sedentary variables were adjusted for time spent wearing the device using the residual method prior to classifying into sex-specific quartiles. Model 1 was adjusted for sex and age. Model 2 was adjusted for sex, age, education, smoking, and family income. Model 3 was additionally adjusted for moderate-to-vigorous physical activity. Model 4 was additionally adjusted for waist circumference.
